# Olfactory modulation of colour working memory: How does citrus-like smell influence the memory of orange colour?

**DOI:** 10.1371/journal.pone.0203876

**Published:** 2018-09-13

**Authors:** Kaori Tamura, Masayuki Hamakawa, Tsuyoshi Okamoto

**Affiliations:** 1 Faculty of Arts and Science, Kyushu University, Nishi-ku, Fukuoka, Japan; 2 Graduate School of Systems Life Sciences, Kyushu University, Nishi-ku, Fukuoka, Japan; Universite de Lyon, FRANCE

## Abstract

Olfactory modulation of vision is not well understood whereas visual modulation of olfaction has been more fully investigated. This study aimed to reveal in a simple manner whether there is olfactory modulation of colour working memory using an odour that induces a citrus-like smell and is associated with orange colours. We assumed that the odour would have modulatory effects on the colour information stored in working memory. To clarify whether these effects are supportive or disruptive, during the colour working memory task we measured an event-related potential component, P3, which is involved in attentional processes of working memory. The results indicated that odour presentation mediated a decline in the rate of correct guesses for orange colours. Furthermore, the odour suppressed P3 during reddish-colour retrieval, including orange. These results suggest that colour working memory in orange can be disrupted via olfactory modulation with citrus-like odours.

## Introduction

Humans regularly use information perceived from multiple senses, and sometimes information received from one sense can override or modulate the information received from another. Numerous studies have focused on such multimodal processing, particularly the relationship between visual and olfactory sensations. Vision and olfaction differ in a number of properties. For example, visual sensations have high spatial resolution, but olfactory sensations do not [1]. Meanwhile, a dominant feature of olfaction is that it can influence emotion or evoke memories directly because it is processed in limbic areas, the brain regions underlying emotion, olfaction, and memory, among others. Indeed, in some circumstances olfactory sensation can augment the impressions of visual images in daily life. For example, some odours can make a food image more attractive, while an unpleasant odour may make a person look disgusted [2–4]. These effects can occur even if the odour intensities are relatively weak. Similar effects can be induced with some colours and odours where there is a specific correspondence between the two (*e*.*g*. citrus odour and orange or yellow colour) [5,6]. Investigations of multimodal modulation of visual information from olfactory inputs have been conducted in an attempt to understand this phenomenon.

Although numerous investigations have studied visual modulation of olfaction, the opposite effect of olfaction on visual processes has not been studied in detail. Several neuroimaging studies have reported that visual stimuli support accurate olfactory identification when an odour and visual cue (picture or word) are semantically related [7–9]. A similar phenomenon has been observed with chromatic stimuli in both neuroimaging [10] and behavioural studies [6,11–14]. In contrast, olfactory modulation of visual perception has not been fully elucidated especially in neuronal studies. Some reports have shown specific neuronal responses to visual objects associated with semantically-related odours using event-related potential analyses, *i*.*e*. larger N1 evoked by the enhancement of matched visual objects with an odour [12] and N400 evoked by visual priming [15,16]. Furthermore, several studies have reported that recognition of emotional pictures or facial expressions could be modulated by an odour impression [2–4,17,18]. These results suggest that olfactory inputs can modulate visual information when these inputs are associated. Conversely, other reports have shown that some olfactory inputs can modulate reaction times for visual attention and cognitive control tasks, although the odours did not relate to the visual targets [19–22]. These studies support the idea that odours can disturb attention in a general way by exerting any kind of cognitive load. This may indicate that olfactory inputs simply monopolise attentional resources but do not directly modulate sensations. Summarising these studies, it is less known whether there is a modulatory effect of olfactory inputs on processes of working memory for congruent visual stimuli. Furthermore, an important issue is in which direction(s) modulation may occur between processing of visual working memory and odours.

Olfactory perception can vary depending on various factors. It is known that both innate and acquired factors induce individual differences in olfactory perception. For example, genetic variation in olfactory receptors can lead to pleasant sensations (“sweet” and “floral”) for a certain person or unpleasant (“sweaty” and “urinous”) for another when they smell the same odorant [23]. It was also reported that olfactory experience modifies the ratings of olfactory pleasantness [24]. The diversity of olfactory perception among individuals makes it more difficult to uniformly investigate the correspondence between olfactory and visual stimuli among individuals. In addition, perfect verbalization of olfactory perception is impossible [25]. This limitation complicates the investigation of olfactory-visual associations. Actually, it was reported that the association between an odour and a colour varies according to individual cultural background [26–29] or synaesthesia of visual and olfaction [30]. As a first step to understanding the neural mechanism underlying olfactory modulation on the visual cognitive process, a commonly associated olfactory-visual pair would need to be assessed, such as an citrus-like odour and the orange colour [5,6].

It is known that visual information can override olfactory information when there are conflicting inputs from the nose and eyes [1]. This makes it more difficult to investigate olfactory modulation of visual perception. We can predict that selective attention and cognitive load would be mainly allocated to vision and less to olfaction when the frontal cortex receives simultaneous visual and olfactory inputs from a common external object. Potentially, olfactory inputs may not be engaging or attractive enough to influence vision.

However, even if olfaction does not modulate visual inputs when they are presented simultaneously, it may have some influence over visual information stored in short-term memory. The olfactory pathway is unique among the senses in that it is directly linked to the limbic system, which is involved in olfaction and memory. Recent studies have also discussed the role of the limbic system in working memory processes [31,32]. Additionally, it was reported that olfactory inputs disrupted working memory in a rat study [33]. From these results, we assume that olfactory inputs can modulate visual information stored in working memory via the limbic system.

Here, we examine whether an odour that evokes a specific colour can modulate visual information regarding the associated colour stored in working memory. Our study had two aims: 1) to assess whether olfaction might influence visual working memory and 2) to clarify the effect of any modulation as supportive or disruptive. Even if there is modulation of visual memory by olfaction, the specific contribution of the modulation cannot be predicted.

To accomplish our aims in a simple manner, we used a citrus-like odour, and examined both behavioural and neuronal responses to modulation. For the behavioural examination, we conducted a delayed estimation task with colour targets [34,35]. This task measures the capacity of working memory [34,35], and previous visual studies have revealed that there is variation in memory capacity for different colour hues [36,37]. We set up four colour groups (pink, orange, green, and blue) and presented an odorant that evoked a citrus-like odour. We used decanal as an odorant, which was an odour-active compound of mandarin orange [38] and orange sweet oil [39], and induces citrus-like smells according to several standard olfactory databases (see Material and methods). Previous research has proposed a model-fitting method using response errors to produce measures of the probability of information being present in memory and memory precision [35]. This model enabled us to investigate modulation processes induced by olfaction on the retrieval of colour memory. For the neuronal examination, we measured event-related potentials (ERP) after each retrieval cue. ERP components are widely used to investigate initial responses on the millisecond time scale, which cannot be detected by behavioural responses for working memory tasks. This study focused on P3 because anterior P3 has been well studied as a marker of initial attention in working memory tasks [40,41]. Therefore, analysis of P3 will support our understanding of olfactory modulation of visual working memory.

## Material and methods

The current experiment was approved by the ethics committee of Kyushu University. All procedures were performed in accordance with approved guidelines of the ethics committee of Kyushu University. All participants gave written informed consent in accordance with the Declaration of Helsinki before participating.

### Participants

We recruited 20 students from Kyushu University to take part in the working memory experiment (female, n = 10; male, n = 10; mean age ± SD = 20 ± 1.7 years). All participants were without colour vision deficiencies, as assessed by Ishihara’s test, and we confirmed that no participant had any olfactory or neurological deficits based on self-reports.

During paired t-tests for behavioural parameters in the working memory task, we excluded three participants following an outlier analysis with jackknife distances based on multivariate statistics (n = 17 for parameters in the working memory). For the ERP analysis, we analysed the data of all participants with two-way ANOVA (n = 20 for ERP in the working memory) because there were no outliers identified by the outlier analysis.

### Colour and display settings

We used 20 colours for encoding during the task. These colours were determined within a circle in the CIEL*C*h colour space, with a centre space at L* = 70, C* = 43, h = 62 and with a radius of 60 [35], such that all brightness and chroma values were common (L* = 70, C* = 103). The colour set included four colour-hue groups (orange, green, blue, and pink), each consisting of five colour phases equally spaced by 10 degrees. Furthermore, each colour-hue group was equally spaced by 90 degrees of hue angle on the CIEL*C*h circle. The precise colour settings in the CIEL*C*h colour space are summarised in the Supplementary Information (S1 Table). Participants did not know which colours would appear during each task before participation.

All visual stimuli were presented on a calibrated CRT display (MT852-12, Iiyama Corporation, Tokyo, Japan) using Psychtoolbox-3 [42,43] programmed in Matlab (MathWorks, Inc. Natick, MA, USA). The monitor was calibrated once a month following standard methods [44] using a Chroma Meter (CS150, KONICA MINOLTA, INC. Tokyo, Japan). Each coloured square in the sample array subtended 1.7° × 1.7° of visual angle. Each square was centred 8.4° to the left, right, up, and down of fixation.

### Odour preparation and presentation

For odour presentation, we used decanal (CAS: 112-31-2, Tokyo Chemical Industry Co., Ltd., Tokyo, Japan). The olfactory descriptions elicited by decanal were obtained from three of the standard databases, *Sigma Aldrich Ingredients Catalog*: *Flavors & Fragrances* (http://www.sigmaaldrich.com), *Flavornet* (http://www.flavornet.org), and *The Good Scents Company Information System for Aromatic Ingredients* (http://www.thegoodscentscompany.com). Each database provided 3–5 olfactory notes for decanal. From these notes, two olfactory notes (citrus and orange peel) were determined as the olfactory description of decanal because they were common descriptions for decanal in two or more standard databases.

For the experiment, 10 ml of 0.1% decanal (vol./vol.) was freshly prepared with squalene oil (Wako Pure Chemical Industries, Ltd., Osaka, Japan). The odour sample was contained in a vial, which was placed in a water bath kept at 35°C during a session. The water bath was placed in a box with a hole, and the hole was set under the nose of each participant. After the working memory experiment, the unpleasantness of the presented odour was rated on separate visual analogue scales from −3 (very pleasant) to 3 (very unpleasant) (mean ± SEM = 0.55 ± 0.24). We also measured the intensity of the odour using separate visual analogue scales from −3 (very weak) to 3 (very strong) (mean ± SEM = 0.35 ± 0.36) after the working memory experiment.

### Study design and procedures of the working memory task

**Fig 1:**
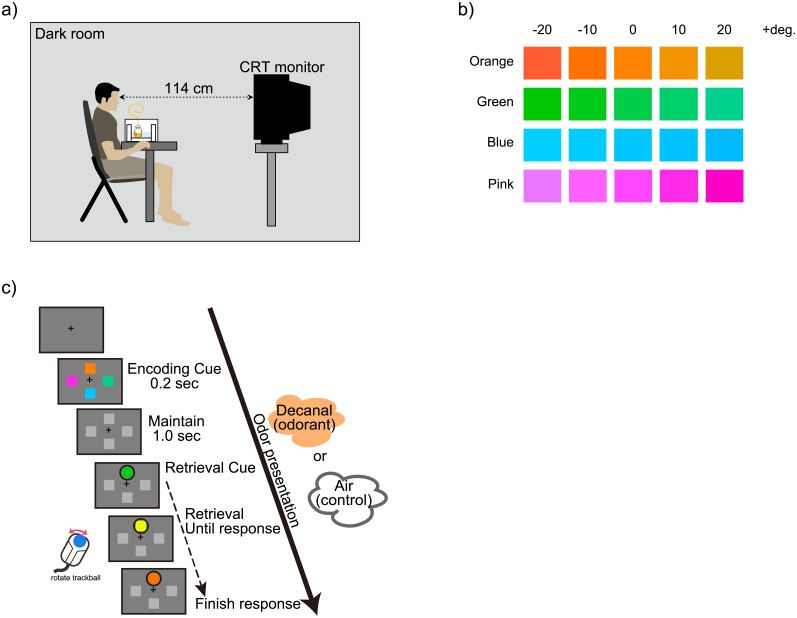
Experimental procedure. (a) The experimental environment is illustrated. (b) An example of colour working memory trials in the control and odorant conditions. (c) The colour stimuli set used in the working memory task. The colours differed by 10 degrees each horizontally, and 90 degrees each vertically in the CIEL*C*h colour space.

Participants performed a colour working memory task in a dark room under two conditions (control and odorant), the order of which was randomised (Fig 1). In the odorant condition, we odorised the room using 0.1% decanal (vol./vol.) during the session. In the control condition, solvent (squalene) was used instead of decanal. To avoid a carry-over effect of the odour, participants rested for approximately 5 minutes between the control and the odorant conditions. Before and after each session, the experimental room was deodorised using a deodoriser (DAS-15D, FUJITSU GENERAL, Tokyo, Japan). The effect of deodorizing was confirmed without the working memory task (see S1 Fig and S1 Method). The deodorizing evaluation was tested in the same environment with the working memory experiment (S1 Fig), where participants were asked to evaluate the odour intensity. All participants judged that there was no odour after deodorizing for 5 min except for 2 participants whose noses were congested (S2 Table).

Colour working memory was measured using a modified delayed estimation paradigm [34]. We developed the program for our procedure using Psychtoolbox [42,43] based on an open sample code [45] (http://bradylab.ucsd.edu/matlab.html). In the colour working memory task, four squares filled with colours were presented for 200 ms in each trial. The colour of each square was selected from each colour group (see Colour and display settings), and the display background was 50% grey, defined by Psychtoolbox-3 [42,43]. The participants were instructed to remember the colour of all four squares. Subsequently, the squares were filled with grey of the same brightness level as the presented colours (CIEL*C*h, L* = 70, C* = 0.0, h = 0.0) for 1000 ms. Memory for the colours was then tested one at a time in a randomly chosen sequence. Only two squares were tested in each trial, such that the participants were asked to respond to two colours out of four in each encoding. Each session included 120 trials, and the participants were tested on 240 colours in a session. We regarded the response order (first and second responses) in each trial as the independent factor. The order of colours was pseudo-random.

At the beginning of each retrieval phase, a cursor and a black circle appeared around a square chosen at random. The selected square was filled with a colour with a randomly selected hue value and the same levels of brightness and chroma as the previously presented colour (CIEL*C*h, L* = 70, C* = 103). Participants rotated a trackball to move a cursor; the angle between the cursor and the centre of the circle determined the hue value of the square, and the colour was changed according to the movement of the cursor. When they decided that the current colour was correct, they clicked a button on the trackball. Subsequently, another circle appeared around another square, and the participants responded in the same way. The locations of the squares to which participants responded were selected in a pseudo-random order. The angular error (answered angle minus correct angle) was regarded as a measure of accuracy.

### Model fitting analysis

We obtained 240 angular errors in each session for each participant, as participants responded to two of the target colours. Thus, we separated the angular errors according to condition (control or odorant), colour-hue group (orange, green, blue, and pink), and participant. Categorised errors provided histograms from −180 to 180° and these histograms were fitted to a mixed model using a uniform distribution and a von Mises distribution (normal distribution for circular data) [35]:

$$p(YX)\;=\;P_{m}\times \text{von}\;\text{Mises}(Y;\mu ,\text{SD})+(1-P_{m})\times \frac{1}{2\pi },$$ (1)

where Y denotes angular errors in stimulus X. This model fitting estimated two parameters: P_failure_, the height of the uniform distribution and SD, the width of the von Mises distribution. Pm was equal to 1 − P_failure_ and represents the probability that the cued item was present in memory at the time of testing. SD represents the precision of continuous memory. The parameter, μ, which indicates the mean of von Mises, was set to zero [35]. To obtain appropriate parameters from each model fitting, we used Bayesian workflow [46]. In this workflow, we conducted a 15000 Markov Chain Monte Carlo procedure (excluding the first 5000 samples as a burn-in period) to obtain the posterior distribution of each parameter value and define an appropriate value using the maximum of the posterior distribution. Model fitting and parameter selection were performed using MemToolbox [46].

### EEG measurement and analysis

EEG was performed across eight channels with gold active electrodes according to the International 10–20 system (Fz, Cz, Pz, Oz, F3, F4, P3, and P4). Electrooculography (EOG) was recorded across two channels (HEOG and VEOG) at the same time. The reference electrode was placed on the tip of the nose during recording and re-referenced to an average of A1 and A2 offline. The ground electrode was placed on Fpz. Electrode impedances were maintained under 50 kΩ at the beginning of the recordings. EEGs were recorded using a Polymate 2 (AP-216, Digitex Lab. Co. Ltd, Tokyo, Japan), amplified using a 3-s time constant and low-pass 100-Hz filter, and digitalised at a sampling rate of 1000 Hz.

The EEG data were filtered offline using a 0.1−50 Hz FIR band-pass filter and 60 Hz notch filter. Epochs were rejected when EOG exceeded 80 μV within −200 to 1000 ms. Epochs were segmented using the cues (memory cue/retrieval cue), with the stimulus onset set at 0 ms, and then the epochs were averaged for each participant. The mean baseline amplitudes (−200–0 ms) were subtracted from each ERP. Subsequently, we averaged the ERPs for all participants to create a grand average ERP for each channel. For statistical analysis, we analysed the ERP at midline electrodes (Fz, Cz, Pz, and Oz) because the component of interest (P3) is known to typically appear in midline locations [40]. The remaining electrodes were used to confirm the absence of external noise. All preprocessing was performed using self-made programs in Matlab (MathWorks, Inc. Natick, MA, USA).

### Colour association

To confirm the association with colours and the odour, the participants in the working memory task were asked which colour they felt was most associated with the odour. They selected one colour from all the tested colours presented on the same CRT display as that used for the experiment after they completed all working memory tasks. Eight of 20 participants failed to respond with an associated colour because of a system problem.

We further recruited 19 participants to compensate for the loss of data in the colour association questionnaire (see S2 Fig and S2 Method). These participants did not overlap with those who answered the colour association question after the working memory task.

### Statistics

According to our a priori hypothesis that working memory of the associated colours was affected by the odour, we tested the mean difference between the control and odorant conditions of the parameters for each colour-hue group using a paired t-test. Alpha levels of significance were corrected using the Holms-Bonferroni correction.

For ERP analysis, we conducted a three-way ANOVA for within-subject analysis to compare P3 amplitudes (mean amplitudes within 300–500 ms) across the two conditions (control/odorant), four colour-hue groups (orange, green, blue, and pink), and four midline channel locations (Fz, Cz, Pz, and Oz) for all participants. On finding an interaction effect among conditions, colour-hue groups, and channel locations, we performed two-way ANOVAs across the conditions and colour-hue groups for each channel location. For multiple comparisons, we analysed the simple-main effect of the conditions in each colour-hue group.

All statistical tests were performed using JMP 12 (SAS Institute Inc., Cary, NC, USA).

## Results

### Model fitting of memory errors

For the behavioural results from the main working memory experiment, we used a mixed model with the von Mises distribution [35] to compare the angular errors between the conditions in each colour-hue group. This model fitting provides two parameters, Pm and SD (see Material and methods). We fit the model to each condition, each colour-hue group, and each response order, individually.

The results were analysed by response order, first and second, because the second responses showed significantly lower Pm than did the first (t(270) = −5.3, p < 0.001, two-sided Student’s t-test), though there was no significant difference in the response order for SD (t(270) = −0.29, p = 0.61) (S3 Fig). From these results, we separated the dataset by response order for further analysis because there was a large dissociation in performance.

**Fig 2:**
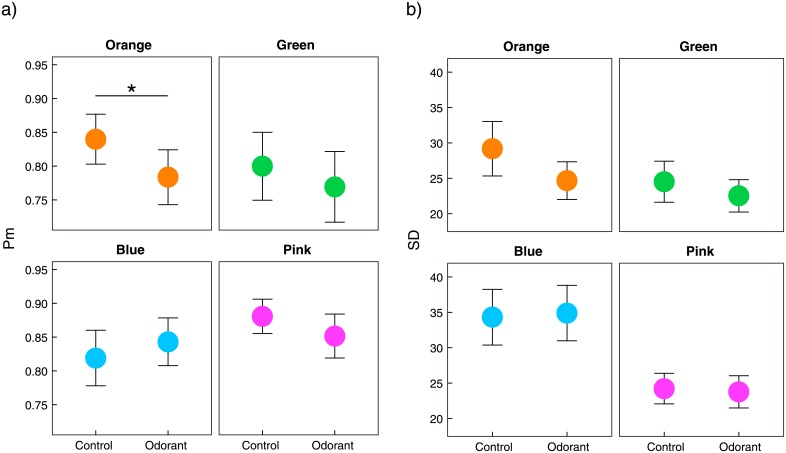
Results of colour working memory task. Mean and standard errors for Pm (a) and SD (b) obtained using model fitting analysis of working memory errors. *: p < 0.05, alpha level corrected using the Holms-Bonferroni method.

According to our hypothesis that the odour could influence memory specifically for the associated colour (*i*.*e*. orange), we applied an *a priori* test to compare the conditions in each colour-hue group for the obtained parameters (Fig 2). We found a significant decrease in Pm for orange memory specifically in the first responses (t(16) = −2.5, p = 0.012, alpha = 0.013, Holms-Bonferroni corrected, paired t-test, control > odorant). However, there were no significant differences in Pm for the other colour-hue groups (−1.1 ≤ t(16) ≤ −0.025, p ≥ 0.16) (Fig 2a). These results indicate that the odour only influenced the probability of a memory being present for orange colours. SD parameters did not show any significant differences for each colour-hue group in the first responses (−1.4 ≤ t(16) ≤ 0.18, p ≥ 0.094, paired t-test, control > odorant) (Fig 2b), indicating that memory precision was not changed by the odorant supplementation. In the second responses, there were no significant differences between the conditions for Pm and SD (Pm: −1.3 ≤ t(16) ≤ −0.24, p ≥ 0.10, SD: −0.72 ≤ t(16) ≤ 1.0, p ≥ 0.24).

### Suppression of P3 by the odour

**Fig 3:**
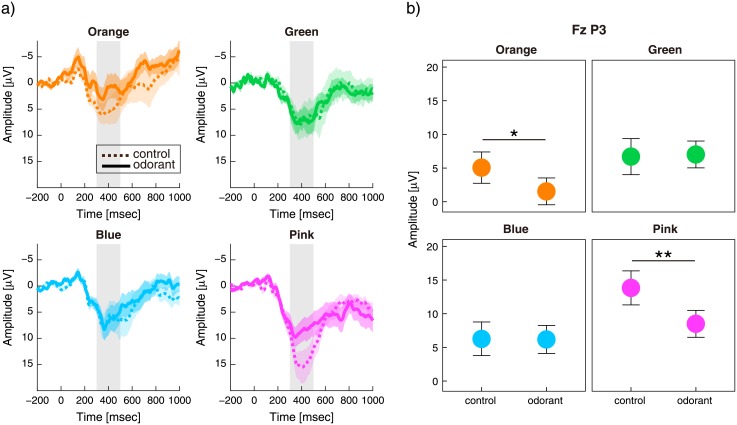
ERP results, including P3 amplitudes. (a) Grand average and standard errors of ERP waveforms after retrieval onset at the Fz electrode. (b) The mean and standard error of P3 amplitudes (300–500 ms) at the Fz electrode. *: p < 0.05, **: p < 0.01, p values were obtained by testing simple main effects.

The ERP waveform was obtained for each condition and each colour-hue group. In each ERP, an obvious positive peak was recognised around 400 ms after the retrieval cue onset (peaks in grey area of Fig 3a and S4 Fig). We suggest that the positive peak falls into the same category as the component P3 determined by its latency and polarity [40].

For neuronal responses, we did not assume any *a priori* hypothesis to examine any interaction effects among the three factors (condition × colour-hue group × channel). We performed a three-way ANOVA for the mean amplitude of the P3 (300–500 ms after the onset), which suggested a significant interaction effect among the conditions, colour-hue groups, and midline channels (F(9,171) = 3.9, p < 0.001).

Subsequently, we separated the dataset according to channel and performed a two-way ANOVA (condition × colour-hue group). We selected the Fz electrode for this analysis because its mean amplitude was largest (mean ± SEM = 6.9 ± 0.83). At the Fz electrode, the main effect of the conditions was marginally significant (F(1,19) = 3.5, p = 0.077) and that of the colour-hue groups was significant (F(3,57) = 12, p < 0.001), with a significant interaction (F(3,57) = 3.9, p = 0.0056).

To investigate the effects of odour presentation, we analysed a simple main effect of the conditions in each colour-hue group. Our results suggested that odour presentation led to a significant decline in P3 amplitudes for orange and pink (orange: F(1,54) = 4.9, p = 0.031; pink: F(1,54) = 11, p = 0.0015) (Fig 3b). There was no significant effect of odour presentation on P3 amplitude in the green and blue groups (green: F(1,54) = 0.041, p = 0.84; blue: F(1,56) = 0.0033, p = 0.95). The amplitudes at other midline electrodes showed a significant main effect of colour-hue group (F(3,57) ≥ 13, p < 0.001), but no significant main effect of the condition (F(1,19) ≤ 1.7, p ≥ 0.21) or interaction effect (F(3,57) ≤ 1.43, p ≥ 0.24).

Multiple comparisons between orange and the other colours within the odorant condition showed that the amplitude of orange was lower than that of the other colours (orange vs. pink: t(19) = 2.9, p = 0.0094, alpha = 0.017; orange vs. green: t(19) = 2.9, p = 0.0099, alpha = 0.025; orange vs. blue: t(19) = 2.5, p = 0.024, alpha = 0.05, two-sided paired t-test, Holms-Bonferroni corrected) (S5 Fig). Multiple comparisons within the control condition showed that the amplitude of pink was significantly larger than that of the other colours (pink vs. blue: t(19) = 4.4, p < 0.001, alpha = 0.017; pink vs. green: t(19) = 4.2, p < 0.001, alpha = 0.025; pink vs. orange: t(19) = 4.2, p < 0.001, alpha = 0.05, two-sided paired t-test, Holms-Bonferroni corrected) (S5 Fig).

P3 is known to have two subcomponents: an early P3 peak in frontal regions (P3a) and a later P3 peak in parietal regions (P3b). Various studies have investigated the relationship between these subcomponents and working memory processes [40]. To specify the area reflecting olfactory modulation, parietal peaks (at Pz) between 300 and 500 ms after stimulus onset were also tested with a two-way ANOVA (condition × colour-hue group). We did not observe any significant main effect of the condition (F(1,19) = 0.45, p = 0.51) or any interaction effect (F(3,57) = 0.32, p = 0.81). However, there was a significant main effect of colour-hue group (F(3,57) = 22, p < 0.001) (S4 Fig). Furthermore, topography plots of the measured channels showed suppression of frontal positivity by the odour, especially in the orange group (S6 Fig).

To investigate the source of the P3 peak, we analysed positivity in other time windows, specifically 250–280 and 250–500 ms after stimulus onset at Fz and Pz. During the early time window (250–280 ms) at Fz, there was no main effect of the condition (F(1,19) = 0.56, p = 0.46) and no interaction effect (F(3,57) = 1.5, p = 0.21). In contrast, during the later time window (250–500 ms) there was a marginal main effect of the condition (F(1,19) = 3.1, p = 0.093) and a significant interaction effect (F(3,57) = 4.1, p = 0.010). Further testing in the later time window showed similar characteristics to the period 300–500 ms after stimulus onset. That is, P3 for orange and pink colours was reduced by odour presentation (orange: F(1,54) = 5.6, p = 0.022; pink: F(1,54) = 8.7, p = 0.0047). For the other colours, there was no significant effect of odour presentation on P3 (F(1,54) ≤ 0.054, p ≥ 0.82). In both time windows, there was a significant main effect of colour-hue group (F(3,57) ≥ 7.9, p ≤ 0.001). At Pz in both time windows, there was no significant main effect of the condition (F(1,19) ≤ 0.41, p ≥ 0.52) and no interaction effect (F(3,57) ≤ 0.47, p ≥ 0.71). Finally, in both time windows, there was a significant main effect of colour-hue group (F(3,57) ≥ 6.5, p ≤ 0.001). In summary, results from the P3 peak suggest that olfactory effects on working memory retrieval are observed in frontal regions.

Although it is difficult to specify areas related to olfactory modulation of working memory from scalp potentials, previous studies have provided several candidate areas and pathways. It is known that the anterior cingulate is the source of the P3a peak, playing a role in controlling attention levels for working memory processes [40]. In addition, responses on the colour working memory task may be related to colour selection processes. Colour selection processes involve the lateral geniculate body and V4, and it has been reported that neuronal activity underlying colour selection at V4 reflects the parietal P1 peak [47]. In the current study, we analysed the P1 peak (150–190 ms) at Pz, but observed no significant main effect of the condition (first response: F(1,19) = 0.28, p = 0.60; second response: F(1,19) = 0.20, p = 0.66) and no interaction effect (first response: F(3,57) = 0.37, p = 0.77; second response: F(3,57) = 1.1, p = 0.34).

We also confirmed the P3 peak in the second response at Fz, but observed no significant main effect of the condition (F(1,19) = 0.077, p = 0.79) and no interaction effect between the conditions and colour-hue groups (F(3,57) = 1.1, p = 0.35). In contrast, there was a significant main effect of colour-hue group (F(3,57) = 12, p < 0.0001) (S7 Fig). Therefore, the results from the second responses did not show any commonalities with those from the first responses.

The ERP waveforms after the onset of the encoding cue were also compared between the control and odorant conditions, but visual inspection suggested no differences in any component (S8 Fig).

### Odour-colour association

The association between decanal and the colours was evaluated. Although the results from the remaining participants showed that orange colours were most associated with decanal (orange: n = 7, green: n = 4, pink: n = 1), there was no significant difference in the ratio of associated colours (Ch^2^(2) = 5.1, p = 0.80).

To compensate for the loss in responses, we further recruited 20 participants who did not participate in the working memory experiment and tested the odour-colour association without the working memory task (see S2 Fig, S2 Method, and S3 Table). We could not find any significant difference in the ratio of associated colours (Ch^2^(3) = 2.8, p = 0.42), though the results from this additional experiment also suggested that the orange colours were most associated with decanal (orange: n = 8, green: n = 3, blue: n = 4, pink: n = 4, one participant was excluded due to colour vision deficiency) (see S3 Table).

## Discussion

In this study, we attempted to clarify the effects of olfactory modulation on visual working memory, in particular the colour associated with an odour. The results showed that the presentation of a citrus-like odour decreased memory-holding characteristics for the orange colour. In addition, there was a relative decline in P3 amplitude associated with odorant presentation during retrieval of the orange and pink colours. In summary, the behavioural and neurophysiological responses suggest that the memory of orange colours was significantly modulated by the odour of decanal. From these results, we suggest two possible conclusions: 1) olfactory modulation of colour working memory exists; and 2) the citrus-like odour resulted in disruptive effects on working memory of the orange colours.

We demonstrated the effect of decanal on the orange colour memory in both behavioural and neuronal responses. Decanal is an odour-active compound of orange sweet oil [39] and mandarin orange [38], and several standard databases (*i*.*e*. *Sigma Aldrich Ingredients Catalog*: *Flavors & Fragrances*, *Flavornet*, and *The Good Scents Company Information System for Aromatic Ingredients*) exhibited that it induces citrus and orange-peel like smells. This citrus-like smell could modulate on working-memory holding characteristics of orange colours, specifically. The mechanism of olfactory modulation has been still controversial, but a candidate of initial pathway has been proposed. Olfactory inputs from olfactory bulb or piriform cortex and visual inputs from retina are converged at olfactory tubercle [48], and the olfactory tubercle projects into orbitofrontal cortex, which was a proposed area to identify the odour and integrate odour and colour information [10,49]. Decanal, the odour-active compound of orange sweet oil could modulate on orange-colours input in this part of the pathway. Generally, individual difference in olfactory perception can complicate the analyses of olfactory-visual associations. Several past studies have shown that olfaction is affected by experience, learning, context, and other factors [50–56]. Indeed, it was reported that the association varies according to factors including cultural background [26]. These studies suggest that the association between an odour and colour would greatly depend on individual background. The present study therefore focused on an olfactory-visual pair, citrus-like odour and orange colour, to interpret the results in a simple and consistent manner. As expected, the citrus-like odour had an effect on the orange colour memory (Fig 3).

The behavioural responses to orange colours were consistent with the neuronal responses, suggesting a modulation effect. In the model fitting of behavioural responses, there were decreases in Pm parameters for the orange colour induced by the odour presentation. Pm has been used to predict the all-or-none probability that a target colour is present in memory [35]. We found that the citrus-like odour decreased orange Pm, *i*.*e*. the probability that orange colours were held in working memory. The neuronal responses during retrieval of memories supported the above behavioural results. During orange colour retrieval, we observed a significant decline in the frontal P3 peak induced by the odour presentation.

There are two potential mechanisms by which the reduced positivity was induced. It is possible that the P3 peak was actually P3a, which is observed at frontal sites around 300 ms after stimulus onset [40]. The P3a peak is a known marker for the allocation of attentional resources [40]. Therefore, we suggest that odours reduced the allocation of attention to retrieval of memories of colours that were most associated with the odour. This assumption is supported by the smaller P3a for orange than for the other colours, as shown by the results of a multiple comparison of odorant conditions (S5 Fig). Given the relationship between P3a and attention processes, it is possible that perception of the citrus-like odour demanded a greater allocation of attention when the target colour was orange-like colour. Therefore, we suggest that there was a relative reduction in the demand to allocate attentional resources to the recall of colours. Lower attention could lead to decreased recall probability, particularly for the associated colours. However, it is worth noting that this interpretation is not sufficient to explain the latency of the positivity peak. The P3a peak should be earlier than P3b, another P3 subcomponent observed in parietal regions [40], but we observed that the presentation of odours did not suppress positivity during an earlier time window, but did at a later time window.

Alternatively, the results could be explained by the relationship between positivity suppression and the time window. The source of the positivity may have been parietal, *i*.*e*. P3b, but the peak was observed in frontal regions. P3b is known to be related to memory access processes in working memory [40], and its amplitude may be decreased by higher memory loads [57–60]. If the observed positivity reflected P3b, positivity to orange colours would be suppressed by decanal due to the larger memory load. During working memory for orange colours, related smell of decanal extracted from citrus fruits can increase the amount of information in the memory because of the relationship between the two, leading to a larger memory load. To confirm whether the source was parietal or frontal, further source analysis with multi-channel EEG recording will be necessary. However, our results are consistent with the hypothesis that the decline in frontal positivity resulted from disruption of working memory retrieval, and the disruption was only induced during memory processes for orange colours by the citrus-like odour.

Model fitting analysis also provides measures of memory precision (SD), but we did not observe any influence of odour presentation on this parameter. Previous studies have proposed that there is variability in memory precision for different colour hues [36,37]. Precision of colour working memory is probably due to hue variations and the fact that the odour was not strong enough to modulate it in this case. Consistent with the two parameters obtained using model fitting, we can assume that the citrus-like odour initially precluded recall of orange colours and decreased the probability of correct recall, but once the participants succeeded in recalling the colours, the odour did not affect memory precision.

The decline of P3 amplitude associated with the odour was also observed in responses to pink colours. The pink colour may inherently have unique characteristics, which induce large P3 peaks in the absence of odorants (S5 Fig). These much higher P3a peaks might be due to a dominance of selective attention with particular colour hues [36,37]. It has been reported that some types of “reddish” colour hold an advantage for selective attention [61], but the regularity between colour hues and heterogeneous attention levels is still controversial. In addition to variable attention, P3 amplitude for pink colours in the odorant condition did not show lower amplitudes than those for green and blue (S5 Fig). This result is not strong enough to support the idea that attention levels for pink were modulated by the same mechanism as for orange colours, rather than the alternative that attention to pink in the control condition was so high that there was an apparent decline in the odour condition. In this study, we chose target colours in a systematic manner (see Material and methods). Nonetheless, the use of pink colours might not be an appropriate choice as a control colour.

Regarding the response order, we found a greater decrease in Pm in the second responses, indicating that the probability that a target colour was present in memory was significantly lower in the second responses than in the first. This result may be related to working memory forgetting effects. It has been reported that the first retrieval of a memory disrupts the second retrieval after encoding several targets [62]. During the retrieval of the first response in our experiment, memories of the other three colours may also have been retrieved, resulting in working memory forgetting in the second response. Furthermore, for the second responses, longer maintenance periods of the memory were necessary because the second retrieval cue appeared after the first. These longer maintenance periods might have led to lower performance. As a result of the lower Pm at baseline (*i*.*e*. the control condition), differences in the modulated response (*i*.*e*. odorant condition) were reduced and may have prevented observation of a significant modulation effect in the second responses.

In this study, we found that the modulation effect was disruptive rather than supportive of visual memory for orange colours although the decanal was an odour-positive compound of orange fruits and oil [38,39]. Previous research has suggested that visual modulation of olfactory perception is unlikely [6–8,10,11]. This inconsistency might reflect differences in the experimental procedures used. Previous studies have used visual perception tasks [6–8,10,11], whereas in this study we investigated modulation of visual information stored in memory using a working memory task. Therefore, the cognitive function under investigation was quite different. Furthermore, it has been known that human olfactory discrimination relies on lexical processes to describe an odour [25] in addition to chemical senses. Congruent visual inputs could support the selection of a correct description of an odour, resulting in improved olfactory discrimination. In contrast, visual working memory is deeply relevant for selective attention processes. It is likely that our experimental procedures, including working memory processes, enabled us to identify a negative influence of olfaction on visual attention and retrieval processes. Further investigations are required to reveal cross-modal effects on working memory processes.

Our findings support the existence of olfactory modulation of colour working memory, and these results should be validated with other colour settings and odour candidates. In this study, to simply assess our hypothesis on whether olfaction can influence visual working memory, decanal was solely used as an citrus-like odour as a first step to understanding the neural mechanism underlying the effect of a typically associated olfactory-visual pair, *i*.*e*. orange-colours and citrus-like odours defined in several standard databases. In this study, we could not conclude on whether the association between the orange-colours and decanal was sufficiently strong. Previous studies that have indicated a clear association between odour and visual stimuli have used specific images and natural mixtures of odorants [7,8,16], *e*.*g*. flower pictures and essential oils. We did not use such stimulus pairs because they could induce strong priming effects, and we would not be able to completely separate the priming effects from the pure association between vision and olfaction. Future study is needed to establish a stronger experimental design to show the strength of the association between colours and odours. The manner of odour presentation might be a candidate of improvement and provide stronger evidence. In our experiment, olfactory stimuli were presented by volatilization in the dark room in a simple manner, in which the breathing and sniffing depended on the voluntary control of each participant (*i*.*e*. participants could refrain from breathing through their nose or sniff less often). In previous olfactory studies, a specialised olfactometer or respiratory training enabled control of the timing and volume of sniffing [63,64]. In the present study, such techniques of odour presentation were not used because we aimed to prevent inducing the dispersion of participants’ attention from the task by perceiving air pressure from the olfactometer or by conducting the sniffing in a trained manner. However, rigorous control of respiration or sniffing should be performed to investigate the olfactory modulation of visual working memory in future study. If the timing and duration of odorant presentations could be controlled, the effects without adaptation might be elucidated as a time-series.

Our results indicated significant effects in both the rate of correct guesses and ERP amplitudes for only orange colours, and orange colours showed a certain level of association with decanal. These results, however, were not sufficiently strong to reject a claim that odour quality did not affect visual working memory, but an odour-floating effect can modulate visual working memory only for orange colours. To assess the sensitivity of visual working memory for orange colours, other odours having no association with orange colours should be investigated on whether they could modulate the memory-holding characteristics and neuronal responses for orange. Furthermore, if working memory of orange colours is not modulated by non-associated odours, other pairs of a colour group and an associated odour should be examined in the same way to confirm whether the phenomenon is general or specific to orange. There are various odour candidates for congruency studies with specific colours, such as the pairs of strawberry smell and red or pink colours and woody smell and green or brown colours. Further analyses for other odour-colour pairs would be needed to comprehensively understand olfactory modulation of colour memory. Colour hue perception is easily affected by differences in luminance or chroma levels and environmental factors, such as contrast with background [65] and stimulus context [66,67]. Future research should investigate more colours to extend our findings to a number of different odour candidates.

Our behavioural and neuronal results lead us to conclude that a citrus-like odour, decanal can cause disruptive modulation of orange colours held in working memory. To our knowledge, this is the first report to observe olfactory modulation of visual working memory in humans.

## Supporting information

S1 Method Method of additional experiment to test the deodorizing. (PDF)

S2 Method Method of additional experiment for odour-colour association task. (PDF)

S1 Fig Outline of the additional experiment to test the deodorizing. The experimental condition was the same as in the ERP experiment.
(PNG)

S2 Fig Outline of the additional experiment for the odour-colour association. The experimental condition was the same as in the ERP experiment.
(PNG)

S3 Fig First and second order of colour memory working task. Mean and standard errors for Pm (a) and SD (b) obtained using model fitting analysis of working memory errors, collapsed in the conditions and the colour-hue groups.
(TIF)

S4 Fig ERP waveforms after retrieval onset for the first responses. Grand average and standard errors of the (a) Cz, (b) Pz, and (c) Oz electrodes are shown.
(TIF)

S5 Fig Mean and standard error of P3 amplitudes (300–500 ms) at the Fz electrode. The data are similar to Fig 3b. *: P < 0.05, **: P < 0.01, two-sided paired t-test, alpha levels corrected using the Holms-Bonferroni method.
(TIF)

S6 Fig Topography plots. The averaged ERP amplitudes from 300–500 ms after stimulus onset for the first responses are shown. The colour bars show amplitude [μV].
(TIF)

S7 Fig ERP waveforms after retrieval onset for the second responses. Grand average and standard errors of the (a) Fz, (b) Cz, (c) Pz, and (d) Oz electrodes are shown.
(TIF)

S8 Fig Grand average waveforms after encoding cues. The mean baseline amplitudes before the encoding onset (−200–0 ms) were subtracted from each ERP.
(TIF)

S1 Table Correspondence between colour label and original CIEL*C*h values. (PDF)

S2 Table Result of the additional experiment to test the deodorizing. (PDF)

S3 Table Result of the additional experiment for odour-colour association. (PDF)
