# Supplementary material for: Olfactory modulation of colour working memory: How does citrus-like smell influence the memory of orange colour?
Source: PLoS One. 2018 Sep 13;13(9):e0203876. doi: 10.1371/journal.pone.0203876 (PMC6136778; doi:10.1371/journal.pone.0203876)
Supplement: S1 Method — (PDF) [file pone.0203876.s001.pdf]

S1. Method of additional experiment to test the deodorizing.

We performed an additional experiment to test whether deodorizing for 5 min can remove the odour. The additional experiment was conducted in the same way with the ERP experiment, as illustrated in S8 Fig. We recruited 8 participants (25 ± 1.5 years, mean ± SEM). They were asked to evaluate the odour intensity on a 6-point scale, from 0 to 5 (0: no odour, 1: extremely weak, 2: weak, 3: normal, 4: strong, 5: extremely strong). The evaluation of the odour intensity was performed three times (before the odour session, after the odour session, and after deodorizing).
