# Supplementary material for: Olfactory modulation of colour working memory: How does citrus-like smell influence the memory of orange colour?
Source: PLoS One. 2018 Sep 13;13(9):e0203876. doi: 10.1371/journal.pone.0203876 (PMC6136778; doi:10.1371/journal.pone.0203876)
Supplement: S2 Method — (PDF) [file pone.0203876.s002.pdf]

S2. Method of additional experiment for odour-colour association task

We performed an additional experiment to test the odour-colour association without the working memory task. The additional experiment was conducted in the same way as the ERP experiment, as illustrated in S2 Fig. Decanal or solvent was presented as in the ERP experiment. The order of the odour and no odour sessions was counterbalanced between the participants. During the sessions, all participants were asked to read an identical book, instead of conducting the working memory task. We recruited 20 participants, and a participant was excluded due to colour vision deficiency assessed by Ishihara's test. Therefore, the data of 19 participants (female: n = 9, male: n = 10, mean ± SEM: 21 ± 0.63 years) were analysed.
