# Supplementary figures and images for: Olfactory modulation of colour working memory: How does citrus-like smell influence the memory of orange colour?
Source: PLoS One. 2018 Sep 13;13(9):e0203876. doi: 10.1371/journal.pone.0203876 (PMC6136778; doi:10.1371/journal.pone.0203876)

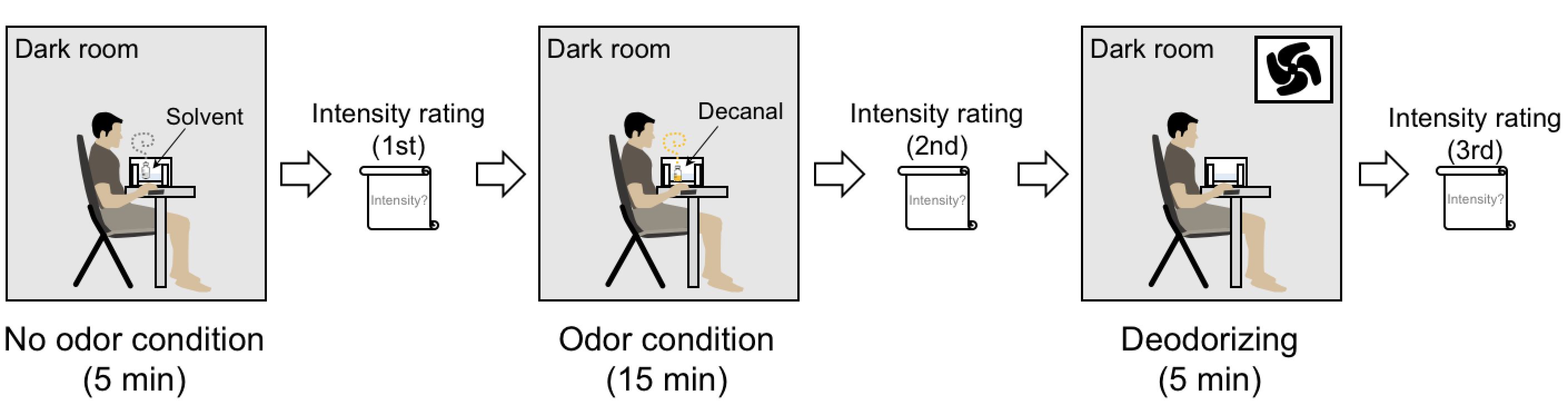

Supplement: S1 Fig — The experimental condition was the same as in the ERP experiment. (PNG) [file pone.0203876.s003.png]

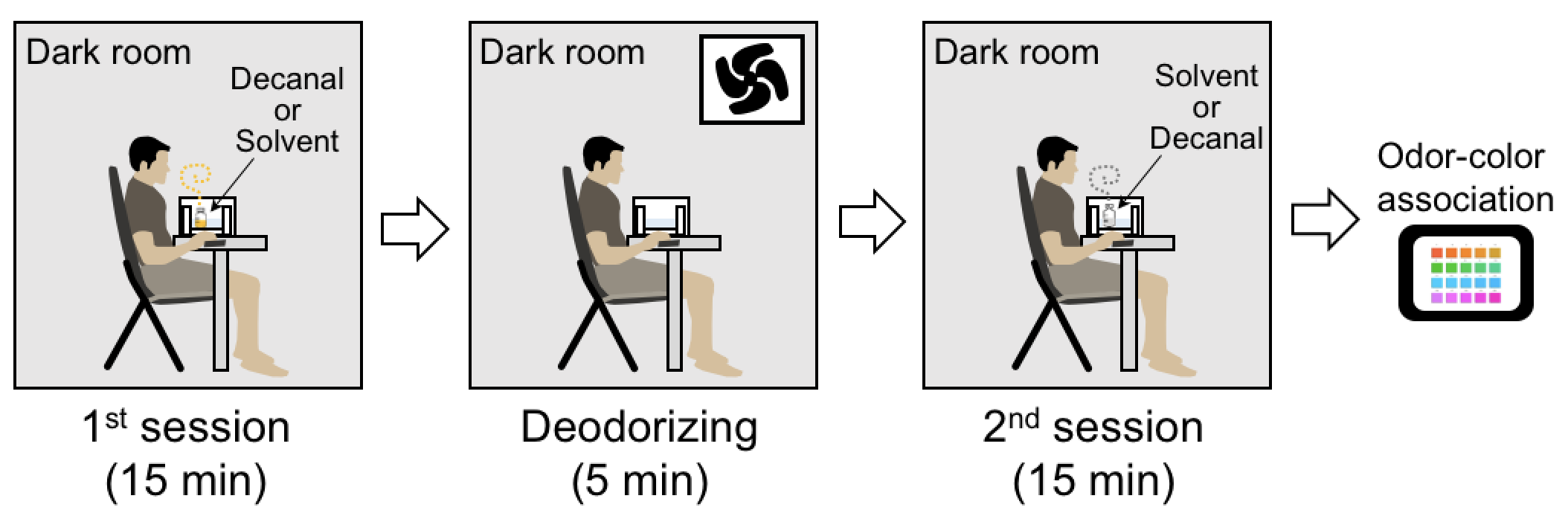

Supplement: S2 Fig — The experimental condition was the same as in the ERP experiment. (PNG) [file pone.0203876.s004.png]

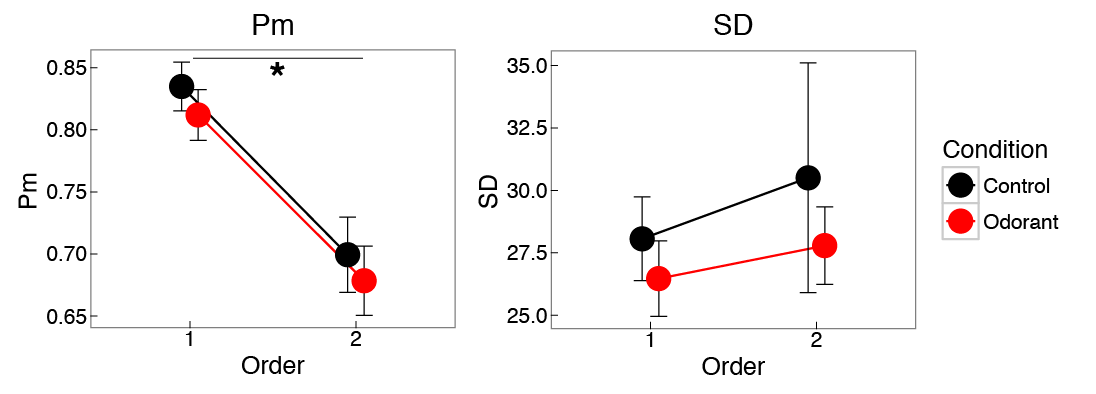

Supplement: S3 Fig — Mean and standard errors for Pm (a) and SD (b) obtained using model fitting analysis of working memory errors, collapsed in the conditions and the colour-hue groups. (TIF) [file pone.0203876.s005.tif]

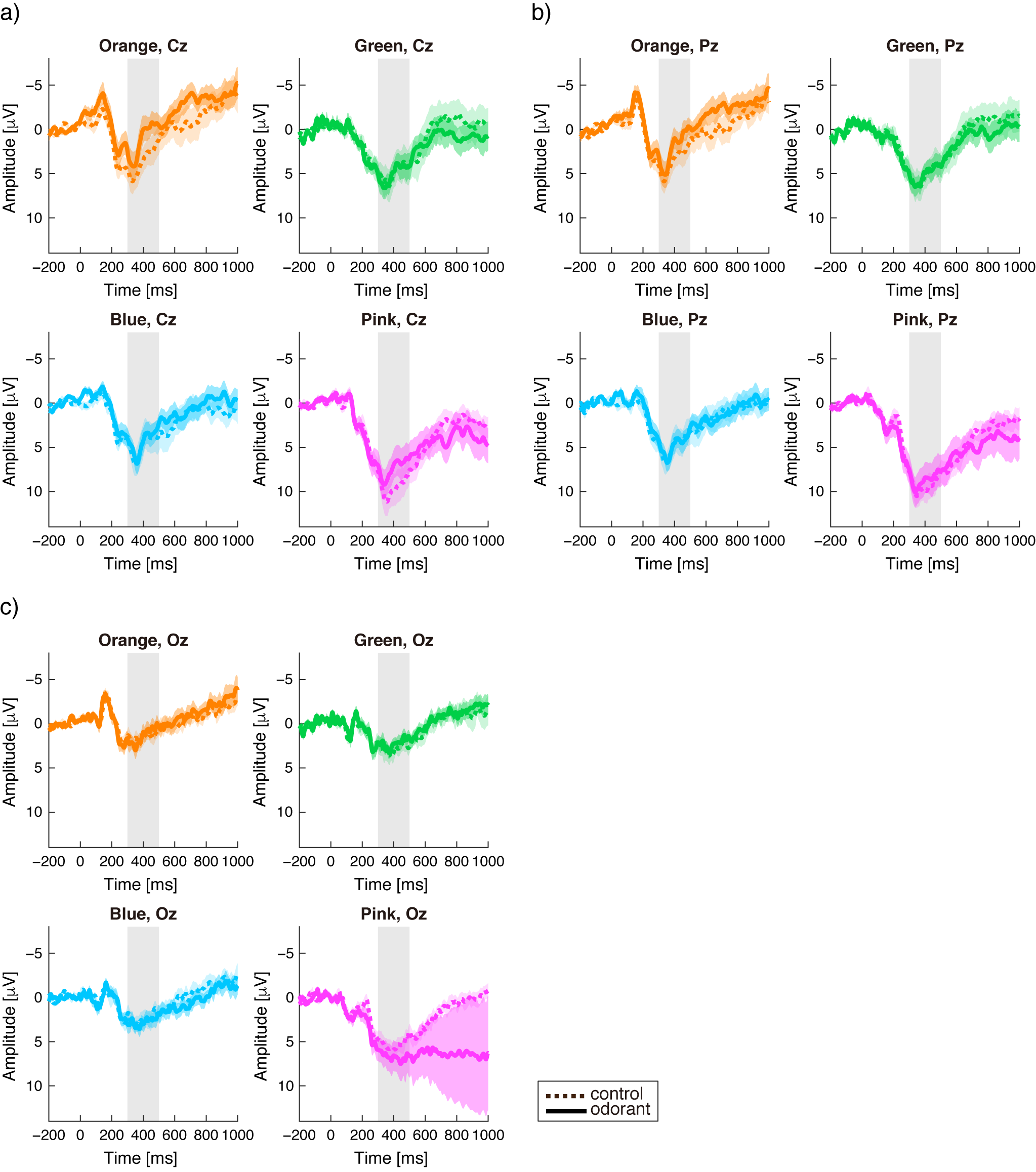

Supplement: S4 Fig — Grand average and standard errors of the (a) Cz, (b) Pz, and (c) Oz electrodes are shown. (TIF) [file pone.0203876.s006.tif]

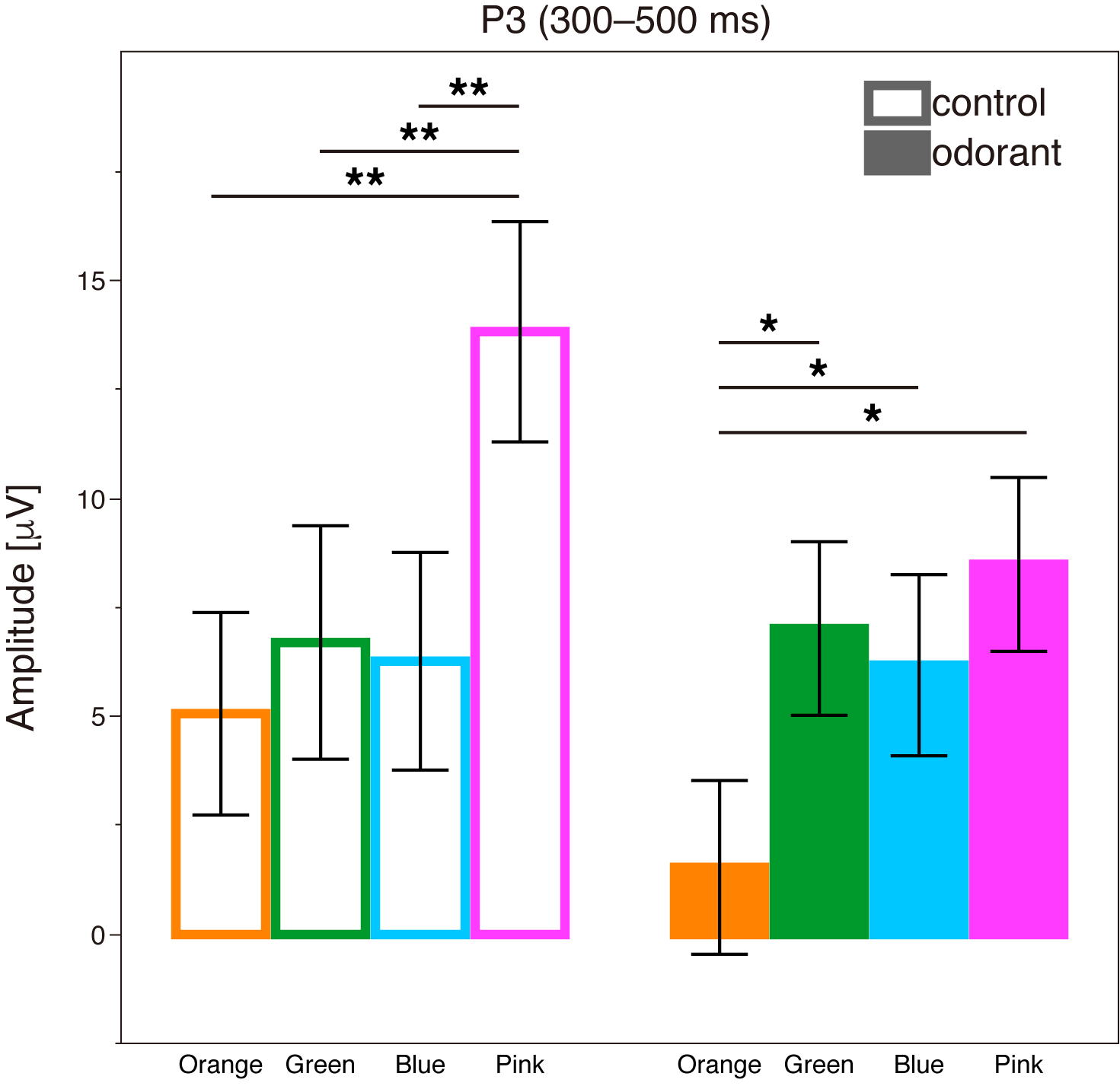

Supplement: S5 Fig — The data are similar to Fig 3b. *: P < 0.05, **: P < 0.01, two-sided paired t-test, alpha levels corrected using the Holms-Bonferroni method. (TIF) [file pone.0203876.s007.tif]

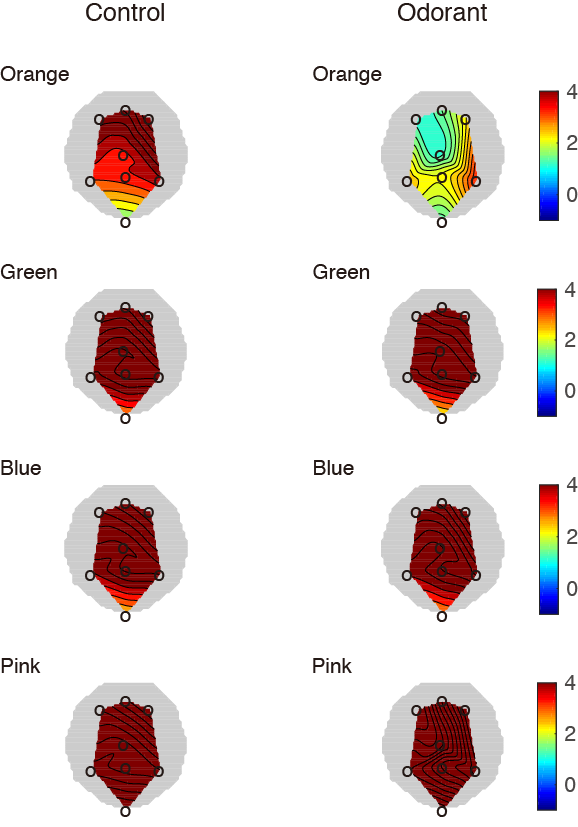

Supplement: S6 Fig — The averaged ERP amplitudes from 300–500 ms after stimulus onset for the first responses are shown. The colour bars show amplitude [μV]. (TIF) [file pone.0203876.s008.tif]

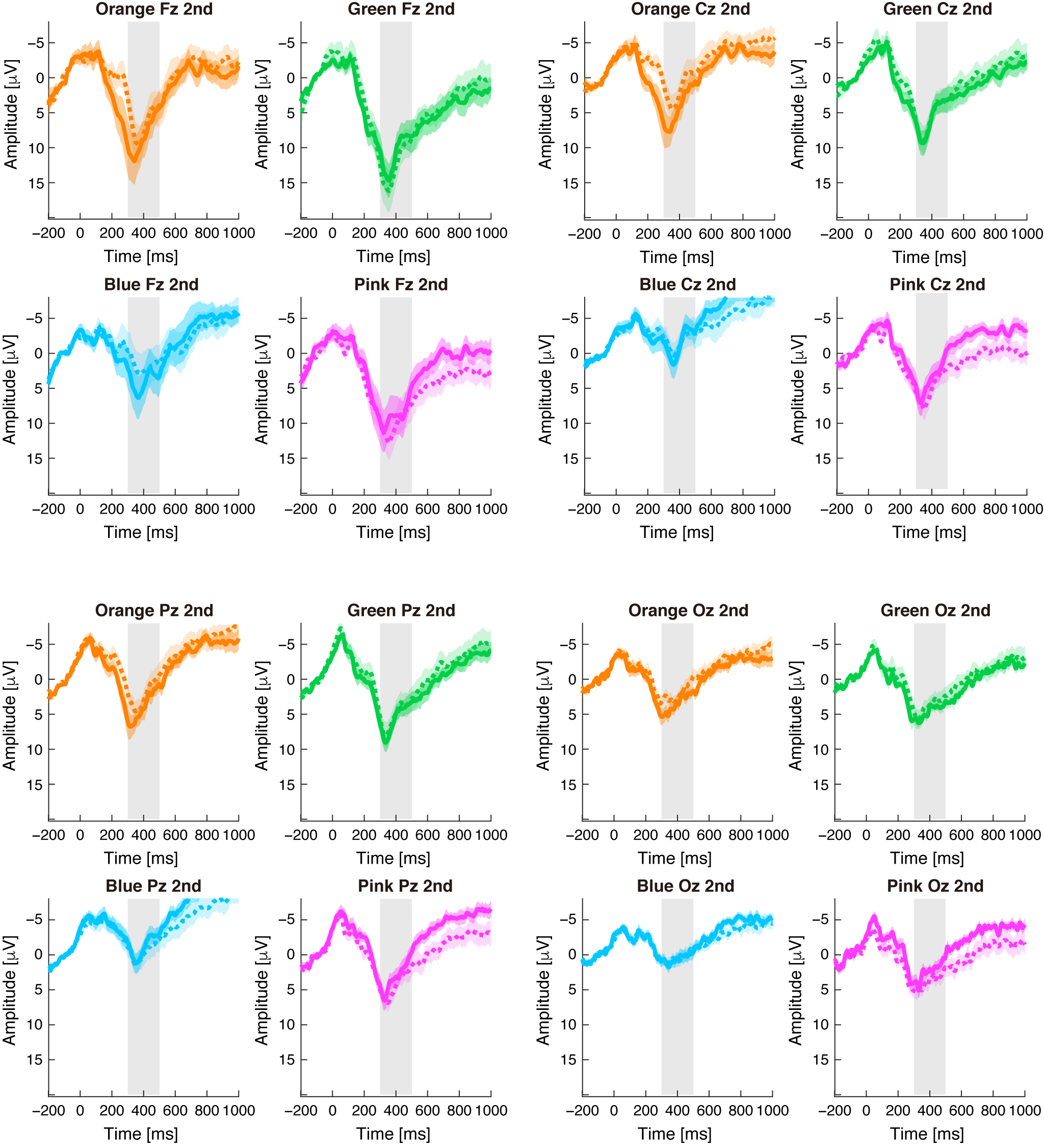

Supplement: S7 Fig — Grand average and standard errors of the (a) Fz, (b) Cz, (c) Pz, and (d) Oz electrodes are shown. (TIF) [file pone.0203876.s009.tif]

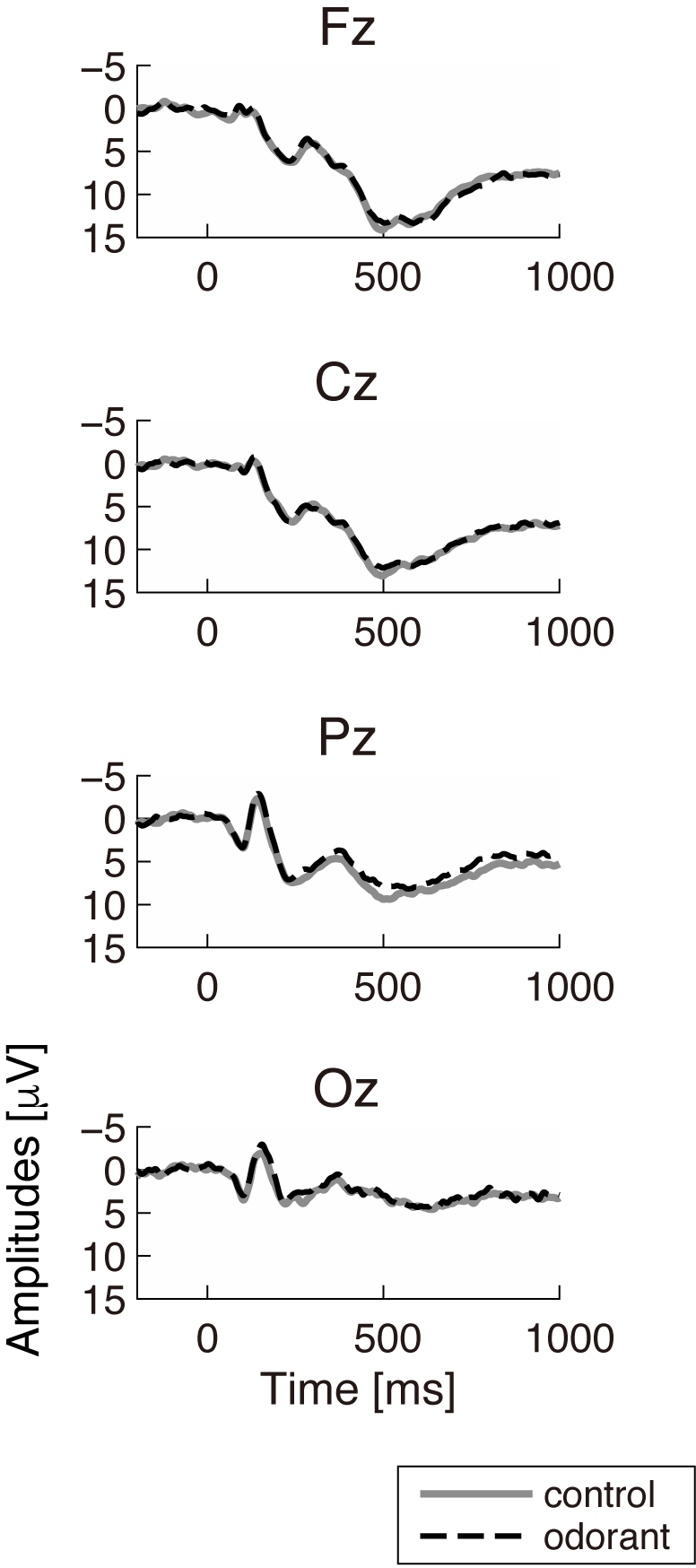

Supplement: S8 Fig — The mean baseline amplitudes before the encoding onset (−200–0 ms) were subtracted from each ERP. (TIF) [file pone.0203876.s010.tif]
