# Supplementary material for: Olfactory modulation of colour working memory: How does citrus-like smell influence the memory of orange colour?
Source: PLoS One. 2018 Sep 13;13(9):e0203876. doi: 10.1371/journal.pone.0203876 (PMC6136778; doi:10.1371/journal.pone.0203876)
Supplement: S1 Table — (PDF) [file pone.0203876.s011.pdf]

Colour label	L*	C*	h
Pink − 20	70	103	312
Pink − 10	70	103	322
Pink 0	70	103	332
Pink 10	70	103	342
Pink 20	70	103	352
Orange − 20	70	103	42.2
Orange − 10	70	103	52.2
Orange 0	70	103	62.2
Orange 10	70	103	72.2
Orange 20	70	103	82.2
Green − 20	70	103	132
Green − 10	70	103	142
Green 0	70	103	152
Green 10	70	103	162
Green 20	70	103	172
Blue − 20	70	103	222
Blue − 10	70	103	232
Blue 0	70	103	242
Blue 10	70	103	252
Blue 20	70	103	262
