# Supplementary material for: Olfactory modulation of colour working memory: How does citrus-like smell influence the memory of orange colour?
Source: PLoS One. 2018 Sep 13;13(9):e0203876. doi: 10.1371/journal.pone.0203876 (PMC6136778; doi:10.1371/journal.pone.0203876)
Supplement: S2 Table — (PDF) [file pone.0203876.s012.pdf]

Participant	Age	Sex	Intensity ratings			Odor-color association	Remarks
			Before odor	After odor	After deodorizing		
1	22	Male	0	2	0	Orange	
2	26	Female	0	4	0	Orange	
3	21	Female	0	4	0	Orange	
4	31	Female	0	3	0	Green	
5	28	Female	0	3	0	Green	
6	22	Male	0	3	0	Pink	
7	22	Male	1	4	3	Green	Self-report as stuffy nose
8	31	Male	2	4	2	Blue	Self-report as stuffy nose
