# Supplementary material for: Olfactory modulation of colour working memory: How does citrus-like smell influence the memory of orange colour?
Source: PLoS One. 2018 Sep 13;13(9):e0203876. doi: 10.1371/journal.pone.0203876 (PMC6136778; doi:10.1371/journal.pone.0203876)
Supplement: S3 Table — (PDF) [file pone.0203876.s013.pdf]

Participants	Sex	Age	Associated colour
1	Female	20	Orange
2	Female	23	Orange
3	Female	29	Orange
4	Male	18	Orange
5	Male	20	Orange
6	Male	21	Orange
7	Male	22	Orange
8	Male	23	Orange
9	Female	18	Green
10	Female	19	Green
11	Male	21	Green
12	Female	18	Blue
13	Female	18	Blue
14	Female	22	Blue
15	Male	21	Blue
16	Female	18	Pink
17	Male	19	Pink
18	Male	22	Pink
19	Male	22	Pink
